# Low‐Cost, Compact Quadrupole Mass Filters with Unity Mass Resolution via Ceramic Resin Vat Photopolymerization

**DOI:** 10.1002/advs.202307665

**Published:** 2023-12-18

**Authors:** Colin C. Eckhoff, Nicholas K. Lubinsky, Luke J. Metzler, Randall E. Pedder, Luis F. Velásquez‐García

**Affiliations:** ^1^ Department of Electrical Engineering and Computer Science Massachusetts Institute of Technology 77 Massachusetts Ave. Cambridge MA 02139 USA; ^2^ Microsystems Technology Laboratories Massachusetts Institute of Technology 77 Massachusetts Ave. Cambridge MA 02139 USA; ^3^ Ardara Technologies L.P. 12941 Route 993 Ardara PA 15615 USA

**Keywords:** additively manufactured mass spectrometry, compact mass spectrometry, glass‐ceramic, quadrupole mass filter, vat photopolymerization

## Abstract

This study reports novel, compact, and additively manufactured quadrupole mass filters (QMFs) with adequate filtering performance for practical mass spectrometry applications. The QMFs are monolithically fabricated via vat photopolymerization of glass‐ceramic resin using 57 µm × 57 µm × 100 µm voxels, and selective electroless plating of nickel‐boron. Experimental characterization of QMF prototypes at 1.74 MHz using FC‐43 yields 131 Da peaks with 0.50 Da full width at half maximum (260 resolution), surpassing the resolution of reported miniaturized counterparts under similar conditions, and being on par with commercial, non‐miniaturized, heavier devices. The sensitivity of the 3D‐printed devices is estimated at 0.13 mA Torr^−1^ (comparable to that of optimized, commercial counterparts), while the devices attained up to 250 Da of mass range (limited by the driving electronics). The work is of interest to low‐cost, capable mass spectrometry, 3D‐printed instruments, and in‐space manufacturing of complex instrumentation.

## Graphical abstract

This study reports novel, compact, and monolithically additively manufactured quadrupole mass filters with filtering performance adequate for mass spectrometry. The devices are fabricated via vat photopolymerization of a glass‐ceramic and selective electroless plating of nickel‐boron. Device operation at 1.74 MHz using FC‐43 yields 131 Da peaks with 0.50 Da FWHM (262 resolution), 0.13 mA Torr^−1^ sensitivity, and 250 Da mass range.

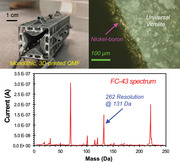

## 1 Introduction

Mass spectrometry (MS) is the gold standard for quantitative chemical analysis, profusely employed in fields such as healthcare, research, and defense.^[^ ^1^ ^]^ The heart of a mass spectrometer is the mass filter, which utilizes electromagnetic fields to sort ionized species in a vacuum based on their mass‐to‐charge ratio. Nonetheless, mainstream mass spectrometers are large, expensive, heavy, and power‐hungry, limiting their use in situ and in autonomous chemical analysis applications. Consequently, there is great interest in developing compact and capable mass spectrometers to address these drawbacks. Although the reported miniaturized mass filters are four orders of magnitude smaller than top‐of‐the‐line devices (Fourier transform ion cyclotron resonance mass spectrometers^[^ ^2^ ^]^), they attain eight orders of magnitude less informing power.^[^ ^3^ ^]^ Therefore, the development of low‐cost, portable MS hardware that is still useful in analytical applications is greatly needed.^[^ ^4^ ^]^

Quadrupole mass filters (QMFs) employ a set of four precisely positioned rods biased at a mix of radiofrequency (RF) and direct current (DC) voltages to create a potential well that transmits only ions of a certain charge‐to‐mass ratio^[^ ^5^ ^]^; by sweeping the amplitude of the RF‐DC voltage mix, a mass spectrum is obtained. QMFs are very popular in MS because of their simple design, their capability to work at reduced vacuum,^[^ ^6^ ^]^ and their versatility to implement very high‐resolution MS hardware, e.g., triple quadrupoles, where the ions filtered by a first quadrupole are fragmented in a second quadrupole, to be analyzed as fragmentation products in a third quadrupole.^[^ ^7^ ^]^ Mainstream QMFs are fabricated via subtractive manufacturing techniques, involving computer‐numerical‐controlled (CNC)‐guided processes like milling, lathing, and polishing to create separate metallic rods; the rods are assembled and positioned using precision, CNC‐machined ceramic components. Subtractive manufacturing poses challenges in achieving optimal QMF performance due to misalignment of the rods, caused by manufacturing tolerances and assembly procedures. In particular, the need to fasten multiple parts together greatly limits the ability of conventional QMFs to be cost‐effective without compromising performance. Furthermore, the great majority of QMFs use circular rods due to cost and ease of manufacturing, although hyperbolic rods are ideally shaped to attain the highest filtering performance.^[^ ^5^ ^]^

Additive manufacturing (AM) offers several advantages that address the limitations posed by subtractive manufacturing. First, AM allows the creation of freeform, complex objects^[^ ^8^, ^9^ ^]^ enabling the use of idealized electrode shapes to improve field quality, while shielding ions from external conductors in a compact design.^[^ ^10^ ^]^ Second, AM facilitates the production of monolithic parts, attaining alignment of the rods without the need for fastening and alignment procedures, simplifying the production process of the QMF, and enhancing rod alignment precision. Third, AM is cost and resource‐efficient at smaller production scales, making it suitable for austere environments with limited manufacturing resources.^[^ ^11^ ^]^ Although AM has some disadvantages, such as potential physical errors due to layer‐based fabrication and comparatively poorer size tolerances and material properties, its capabilities make it a promising approach for developing smaller, lower‐cost quadrupole mass filters with high‐performance capabilities. There are examples of 3D‐printed QMFs, e.g.,^[^ ^12^, ^13^, ^14^ ^]^; however, these devices are not monolithically made and do not perform competitively compared to commercial hardware.

This study presents the design, fabrication, and characterization of novel, additively manufactured, compact QMFs with competitive filtering performance compared to commercial, non‐miniaturized devices, harnessing vat photopolymerization of glass–ceramic resin and surface‐selective electroless nickel‐boron plating. Section 2 describes the synthesized designs, while Section 3 reports the manufacturing aspects of the QMFs. In Section 4, the characterization of the QMFs is presented. Section 5 discusses the results and suggests tentative directions for future research, while Section 6 concludes the work.

## 2 Device Design

A QMF creates a potential well within the space surrounded by its rods. The dynamics of the ions traveling within the potential well are described by the Mathieu equation^[^ ^15^ ^]^

(1) $$\frac{d^{2}u}{d\xi^{2}}+\left[a-2\mathrm{qcos}\left(2\xi \right)\right]u\;=\;0.$$

Whether an ion trajectory is stable within the quadrupole (i.e., whether the ion is transmitted by the QMF) is associated with the value of the associated dimensionless parameters *a* and *q*, given by

(2) $$a\;=\frac{8eU}{mr_{o}^{2}\omega^{2}}\;$$

(3) $$q\;=\frac{4eV}{mr_{o}^{2}\omega^{2}}\;$$

where *U* is the resolving DC voltage, *V* is the zero‐to‐peak amplitude of the AC voltage, *e* is the electron's charge, *m* is the mass of the ion, ω is the angular frequency of the AC voltage (ω  =  2π*f*, where *f* is the frequency of the AC voltage), and *r*_o_ is the inscribed radius (half the separation between opposite rods). For quadrupole mass filters operated in resolving mode in the first stability region of the Mathieu equation (the stability region at which the great majority of QMFs operate), these combined parameters are selected to be just under the tip of the stability diagram, where *a*  =  0.237 and *q*  =  0.706. Through this *a* and *q* parameterization, there can be an infinite combination of dimensions of inscribed radius, RF frequency, and RF and DC voltages that allow transmission for a given mass‐to‐charge ratio. In practice, one selects a combination that provides a compromise between mass range, mass resolution, and absolute sensitivity.^[^ ^16^ ^]^

For this work, the system design was constrained by the maximum device height afforded by the 3D printer (125 mm) and the specifications of the RF electronics available (maximum peak‐to‐peak voltage of 3.6 kV, 1.74 MHz frequency). The voltage level is compatible with standard, vacuum‐compatible SHV‐5 feedthroughs. Given that the ultimate mass resolution is proportional to relative mechanical tolerances,^[^ ^16^ ^]^ the inscribed radius, *r*_0_, was chosen to be as large as possible, to maximize ultimate mass resolution, while allowing for a usable mass range of 250 Da. Using the largest inscribed radius that can be accommodated on the 3D printer platform also maximizes the manufacturing precision of the printed part, as printed objects are discretized in terms of the voxels, and the smaller the size of the voxels compared to the dimensions of the object, the smaller the relative dimensional error. A larger mass range could be effected by reducing the RF frequency (halving the RF frequency would quadruple the mass range), or reducing the inscribed radius (halving the radius would quadruple the mass range), at the expense of ultimate resolution. The mass range could be extended linearly by increasing the available peak RF voltage, but practical considerations of power consumption, quadrupole heating, and vacuum arcing limit the extent of such an increase. Miniaturization of the rod radius could also extend mass range, but at a dramatic decrease in absolute sensitivity, as the absolute ion transmission of a quadrupole is proportional to the inscribed radius to the third and fourth power.^[^ ^17^ ^]^ Therefore, ultra‐miniaturization of quadrupoles results in devices with dubious practical applications. In the cited work, reducing from 20 mm rod diameter to 2 mm rod diameter, with the same exact ionization source and detector, and the same operating conditions, resulted in a reduction in absolute transmission by a factor of 3639.^[^ ^17^ ^]^

The design of the QMFs reported in this study considered two critical aspects, i.e., filter performance and manufacturability. While AM offers the advantage of creating complex geometries not easily attainable via subtractive methods, AM also has inherent limitations that must be carefully considered during the design process of the QMF. In this context, the design features discussed in this section aim to address both manufacturability concerns and the enhancement of filter performance—most design features represent a compromise between these two objectives. The primary focus of the proposed QMF design is attaining a monolithic structure where conductive rods are held by a dielectric structure. This is attained by masking the dielectric parts, electroless plating the full part, and removing the mask.

**Figure 1:**
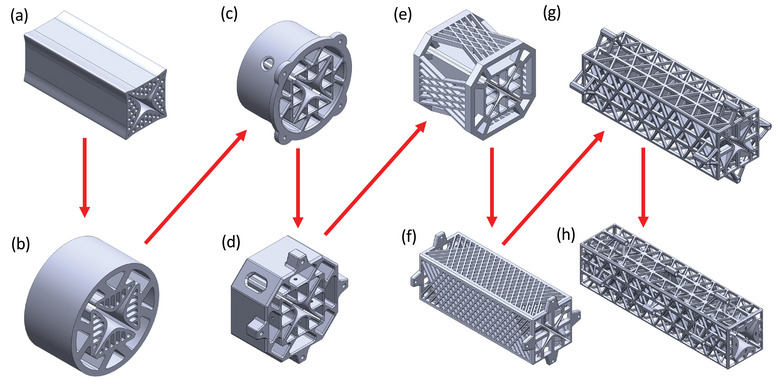
Design evolution of the additively manufactured QMFs reported in this study. a) QMF with extended hyperbolic rods. b–d) QMF designs with cylindrical overstructure. e,f) QMF designs with skeletonized overstructure featuring a rhombic lattice. g,h) QMF designs with skeletonized overstructure featuring a triangular lattice.

Initially, a simple mechanical fusion approach was explored, joining the hyperbolic rods near their asymptotes (**Figure** **1**a). However, this approach proved inadequate, as it led to unavoidable shorting of the rods after metallization. The closely spaced, fused rods posed challenges in masking the connecting material effectively to achieve galvanic isolation of the rods. Furthermore, even if successful, this approach introduced dielectric material directly along the asymptotes of the hyperbolic shapes and near the center axis of the QMF, causing undesirable interference due to charge accumulation during operation. To overcome these limitations, an outer, shell‐like structure encompassing and supporting the rods, referred to in this study as the QMF “*overstructure*,” was explored. Although the overstructure monolithically connects the rods, it is offset from the rods to allow for easier masking and reduced interference with the quadrupolar fields generated by the rods. The overstructure commenced as a solid‐walled shell (Figure 1b–d). Although adequate for quadrupoles of very short lengths (<5 cm), this approach proved challenging to inspect and mask; furthermore, the solid overstructure unnecessarily expends feedstock and adds weight to the part. To address these concerns, a skeletonized overstructure was introduced, first featuring rhombic lattices (Figure 1e,f), and later using equilateral, triangular lattices (Figure 1g–h). This is not a truss structure, as trusses are networks of beams connected by their ends that can only be axially loaded. In the case of the QMF overstructure, the connections between the beams are fully constrained, so bending moments and shear forces are also transmitted. Introducing a skeletonized overstructure not only facilitated easier inspection and masking of the QMF but also significantly improved the strength‐to‐weight ratio of the device. The rods are hyperbolic shells internally supported by a triangular lattice of ribs. Solid infill is not used in the rods to diminish the usage of printable material; having solid infills would also increase the print failure rate due to the higher surface area of each photopolymerized layer.

**Figure 2:**
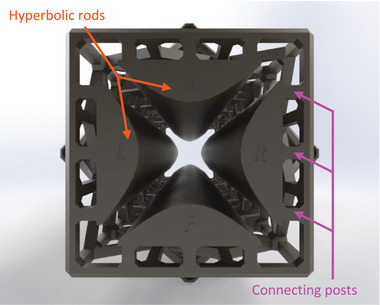
3D CAD of the QMF inlet detailing the connecting posts that attach the hyperbolic rods to the overstructure. The length of the quadrupole is 12.5 cm, while the separation between opposite rods is 12 mm.

Each rod is connected to the overstructure using three small connecting posts positioned at both ends of the rod (**Figure** **2**); this arrangement enables seamless masking of the posts to galvanically isolate the rods from the overstructure while avoiding the presence of dielectric surfaces near the travel path of the ions. The adoption of a skeletonized overstructure sparingly connected to the rods makes it possible to fabricate long QMFs, which benefits the QMF resolution, as it is proportional to the number of motion cycles the ions carry out while traveling through the quadrupole.^[^ ^18^ ^]^ The length of the QMFs developed in this study is 125 mm, i.e., the maximum height of objects that the 3D printer used in this study can create while using posts to attach the part to the printer platform (see Section 3). The distance between opposite rod surfaces of the QMF, i.e., 2*r*_o_, is 12 mm—a value commonly used in commercial QMFs.

**Figure 3:**
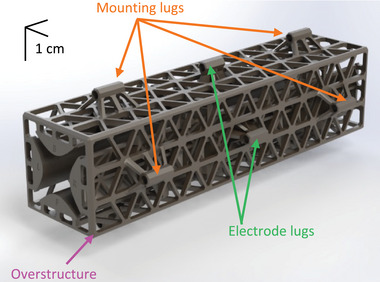
3D CAD render of QMF detailing the electrode and mounting lugs. The length of the quadrupole is 12.5 cm, while the separation between opposite rods is 12 mm.

**Figure 4:**
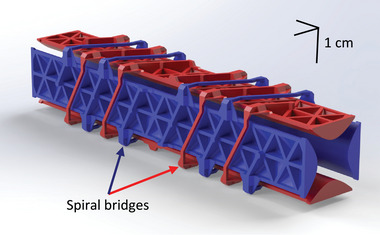
3D CAD render of a QMF sans‐overstructure detailing the spiral bridges that connect opposite electrodes. The length of the electrodes is 12.5 cm, while the separation between opposite rods is 12 mm.

The QMF has a set of lugs to facilitate its interfacing (**Figure** **3**). Near each end of the QMF, there are four lugs that are used to mount the device into an existing mass spectrometry system using 304 stainless steel (SS), CNC machined collars. There are four lugs in the middle of the QMF to enable electrical connection to the rods. The QMF includes a system of spiral bridges featuring alternating paths that either contact the rod or are suspended above it (**Figure** **4**). This arrangement not only creates redundancy in the electrical connections and increases the electrical conductivity between each pair of opposite rods, but it also enhances the structural integrity of the assembly. The spirals act as stabilizing elements, providing increased structural cohesion between rods and improving the overall stability around the center of each rod, where it is most distant from the anchoring posts at its ends that connect to the overstructure. Moreover, the bridges extend along the entire length of the rods to achieve balanced reinforcements, thereby mitigating potential issues related to warping caused by temperature variations, aging, electrical forces, and other factors.

**Figure 5:**
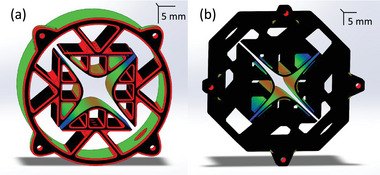
a) 3D CAD render of a curvy QMF. b) 3D CAD render of a polygonal QMF. The length of the electrodes is 2 cm, while the separation between opposite rods is 8 mm.

The quadrupole design is distinctively polygonal (**Figure** **5**), with curvature incorporated only where necessary, e.g., on the rod faces, the screw holes. This choice serves two primary purposes. First, the emphasis on polygonal geometry simplifies the design, making it easier to identify potential manufacturability issues. For instance, using a constant angle chamfer instead of a curved fillet makes it easy to maintain the chamfer angle within the build angle constraints of the printer. In contrast, a fillet would encompass a wider range of angles that can pose challenges for fabrication using a vat photopolymerization printer. Second, the absence of unnecessary curved features results in a substantial reduction in tessellated file sizes. High‐resolution exports of CAD data to a printer can become impractical for complex, curvy designs. By limiting the presence of curves to only where needed, our design allows for seamless export of CAD data at very high resolutions to any post‐processing software, facilitating the subsequent supporting and slicing processes necessary for printing.

## 3 Device Fabrication

The QMFs were made via vat photopolymerization (the dielectric foundation of the devices) and selective electroless plating (electrically conductive surfaces of the devices). Vat photopolymerization was carried out using a Bison 1000 DLP printer (Tethon 3D, Omaha, NE, USA) with a light engine composed of a 1920 × 1080 array of 405 nm UV LED pixels of 57 µm in size. The dielectric structures were printed in 100 µm slices. The vat of the Bison 1000 heats up the resin to improve the adhesion of the printed part to the platform and to reduce the viscosity of the resin during printing. The printer is installed inside a custom, filtered HEPA cabinet to keep a clean and regulated working environment.

The dielectric parts were made with Universal Vitrolite (Tethon 3D, Omaha, NE, USA)—a photocurable glass‐ceramic resin heavily doped with silica microparticles. This printable material is similar to their Bison Vitrolite product, although Universal Vitrolite is significantly less viscous because it has a lower concentration of ceramic microparticles. Ordinarily, components printed with either feedstock are annealed at high temperature (900 °C or more) to fully sublimate the polymer components and sinter the ceramic particles, resulting in a true ceramic part, albeit shrunken. However, prior work with Bison Vitrolite shows that the unfired, as‐printed material exhibits adequate stiffness, chemical resistance, and outgassing for a vacuum analytical application.^[^ ^19^ ^]^ In the same previous study, it was reported that only a portion of the platform of the Bison 1000 could be used to 3D print precision objects using Bison Vitrolite due to the high viscosity of the feedstock and the flexibility of the platform.^[^ ^19^ ^]^

**Figure 6:**
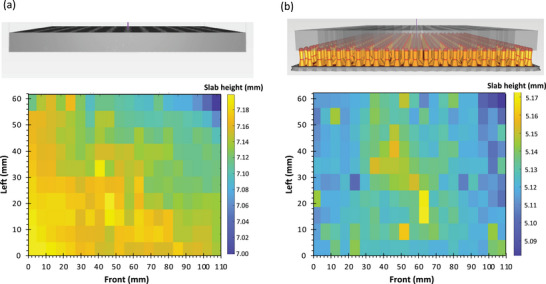
2D metrology field of the height of a) an unsupported slab and b) a supported slab. The standard deviation of the slab height is equal to 31 µm for the unsupported case, and equal to 13 µm for the supported case. The measurements were conducted with a micrometer with 1 µm precision (digital micrometer 293‐340‐30, Mitutoyo America Corporation, Marlborough, MA, USA).

In this study, we found that by supporting the prints using posts at least 5 mm long (instead of printing the objects directly on top of the platform), and by changing the printable material to Universal Vitrolite, it is possible to use the full area of the platform to manufacture precision objects (**Figure** **6**). To the best of our knowledge, this is the first report of a high‐vacuum application using 3D‐printed parts made of Universal Vitrolite. Furthermore, being able to use the full build platform, thereby increasing the size of the precision objects that can be made, is of great interest to the academic and start‐up communities, as the hardware for printing ceramics via vat photopolymerization is significantly cheaper than mainstream, powder‐based printers for ceramics.^[^ ^20^ ^]^

**Figure 7:**
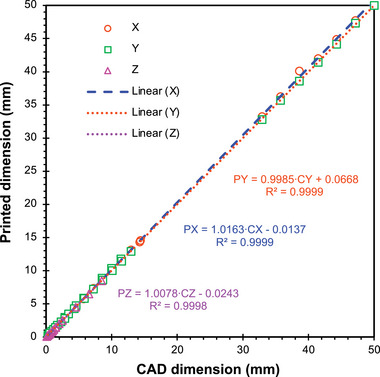
Metrology results from the step pyramids used to benchmark correspondence and linearity between 3D‐printed and CAD features. The linear fits describe the 3D‐printed dimension as a function of the CAD dimension; therefore, for 0 CAD dimension, there is an offset in the printed dimension. Following ISO/ASTM 52900:2015 guidelines, *X* is the in‐plane direction that goes from left to right of the printer, *Y* is the in‐plane direction that goes from back to front of the printer, and *Z* is the out‐of‐plane direction from bottom to top of the printer. In the linear fits, *PY* = printed dimension in *Y*, *PX* = printed dimension in *X*, *PZ* = printed dimension in *Z*, *CY* = CAD dimension in *Y*, *CX* = CAD dimension in *X*, *CZ* = CAD dimension in *Z*.

The linearity and correspondence of the 3D‐printed features made in Universal Vitrolite with respect to the corresponding CAD features were benchmarked by conducting metrology on step pyramids. The width of each step of the pyramid is a multiple of 25 pixels (each pixel measures 57 µm in each in‐plane direction). Similarly, the height of each step pyramid is a multiple of the slice height (100 µm); specifically, the lower ten steps were 2 mm tall (20 layers), while the upper ten steps were 500 µm tall (five layers). Characterization of the small features up to 15 mm was performed with a laser scanning confocal microscope with nanometer‐level resolution (Keyence VK‐X250, Keyence, Itasca, IL, USA). The larger features were measured with a micrometer with 1 µm precision (digital micrometer 293‐340‐30, Mitutoyo America Corporation, Marlborough, MA, USA). The summary of the metrology of the corresponding printed dimensions versus the CAD design dimensions is shown in **Figure** **7**; each measurement is an average of ten samples, with a standard deviation of 24 µm in XY and 10 µm in Z. The linear fits demonstrate excellent agreement and linearity between the 3D‐printed dimensions and the CAD dimensions. The 3D‐printed QMFs are discretized in terms of the voxels (57 µm × 57 µm × 100 µm for this printer); this dimensional precision is at least an order of magnitude coarser than that of the precision machining used in commercial quadrupoles (a fraction of a thousandth of an inch). Improving the manufacturing precision of the devices should yield better device performance. However, this optimization is not essential to conduct the intended proof‐of‐concept demonstration because the current 3D‐printed quadrupoles generate good enough peaks to conduct mass spectrometry with adequate resolution (see Section 5).

**Figure 8:**
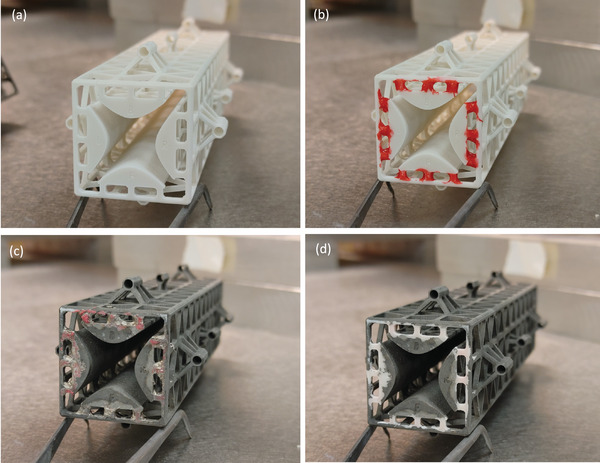
Selected images of a QMF reported in this study during its post‐printing fabrication process. a) 3D‐printed QMF body. b) Masking of the posts connecting the QMF rods and the overstructure. c) Plated QMF with lacquer mask on. d) Plated QMF without masking, yielding a working device. The separation across opposite rods is equal to 12 mm and the length of the quadrupoles is 12.5 cm.

Following the printing process (**Figure** **8**a), the fabricated parts are selectively masked using Procoat maskant lacquer (Maskcoat, West Monroe, LA, USA) to achieve galvanic isolation between the electrode surfaces (Figure 8b). Subsequently, the masked parts undergo electroless nickel‐boron plating (Figure 8c), providing a uniform metallic thin film across all unmasked surfaces.^[^ ^21^ ^]^ Electroless plating was chosen in this study to metalize the rods due to its ability to produce an even and consistent, spatially uniform metallic coating compared to other methods like electrolytic plating or sputtering. This advantage arises from the autocatalytic chemical reactions governing the film deposition in electroless plating (assuming uniform heating and agitation), rather than relying on electric current density or surface exposure. Thicker films are deposited the longer the part is submerged, until the bath is depleted of nickel‐boron, requiring replenishment.

Nickel–boron was selected as the plating material for its exceptional corrosion resistance, ease of plating process, and acceptable electrical conductivity. Moreover, it offers superior corrosion resistance and conductivity in comparison to popular nickel–phosphorus coatings. Despite nickel's high magnetic permeability, which may pose challenges in admitting high‐frequency power signals due to its severe skin effect (≈6 µm skin depth at 1 MHz), this does not significantly impact the performance of the QMF, as commercial QMF rods are typically made of SS, which is significantly less electrically conductive. Upon completion of the plating process, the maskant is removed using a solvent (Figure 8d), revealing the intended bare dielectric surfaces in specific regions (i.e., the posts connecting the rods to the overstructure). This way, it is possible to assign independent bias voltages to each set of opposing rods and to the overstructure. The QMFs have a capacitance of ≈40 pF (BK Precision Model 895 Bench LCR Meter, B&K Precision Corporation, Yorba Linda, CA, USA), while the end‐to‐end resistance of a nickel–boron quadrupole rod measures less than 1 Ω at a frequency of 1 MHz (BK Precision Model 895 Bench LCR Meter, B&K Precision Corporation, Yorba Linda, CA, USA), evidencing the good electrical conductivity of the rods.

The reported 3D‐printed quadrupoles are more compact and lighter than their commercial counterparts. For example, the effective rod diameter of our quadrupole is 12 mm, but the part fits in the installation footprint of a commercial quadrupole with 9 mm diameter rods (in other words, the quadrupoles can be swapped without requiring interfacing parts). Also, the skeletonized design and use of a glass ceramic as structural material makes the 3D‐printed quadrupoles significantly lighter than the commercial counterparts (the density of green, 3D‐printed Vitrolite is ≈1800 kg m^−3^—less than a fourth the density of SS).

## 4 Device Characterization

**Figure 9:**
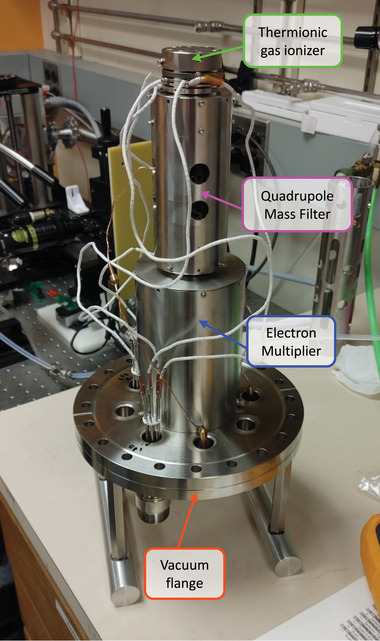
Assembly of QMF, thermionic ionizer, and electron multiplier connected to an 8‐inch UHV vacuum flange.

The 3D‐printed mass filters reported in this study were characterized using a commercial thermionic electron impact gas ionizer (Ardara Technologies Slimline, Ardara Technologies LP, Ardara, PA, USA) and a continuous dynode electron multiplier (Ardara Technologies LP, Ardara, PA, USA) (**Figure** **9**). The components were connected to a flange, which in turn attaches to a vacuum chamber (Kurt Lesker, Jefferson Hills, PA, USA) pumped down by a dry rough pump (Adixen ACP 15, Pfeiffer, Annecy, France) and a turbomolecular pump (HiPace 300 Turbo TC400, Pfeiffer Vacuum Inc., Nashua, NH, USA). The base pressure of the vacuum chamber is ≈1 × 10^−8^ Torr and reaches pressures ≈1 × 10^−5^ Torr when either Argon or perfluorotributylamine (FC‐43) is introduced.

During the experiments, electrons generated by a thermionic emitter collide with the background gas molecules, leading to the creation of ions via fragmentation; the ions are then fed to the QMF. The QMF is biased at a given mix of AC and DC voltages; only ions with a specific mass‐to‐charge ratio are transmitted by the QMF to the electron multiplier, where the current is amplified, and then further processed through a preamplifier to enhance its sensitivity (Ardara Technologies LP, Ardara, PA, USA), and subsequently digitized for further analysis and data recording. By varying the magnitude of the mix of voltages, the QMF can scan a mass range; mass spectra are generated by correlating the ion current collected with the QMF's corresponding magnitude of the mix of voltages (the current is proportional to the abundance of ions with a specific charge‐to‐mass ratio, and the magnitude of the voltages are directly related to the mass of the ion, assuming the ions are singly ionized, as expressed by Equations (2) and (3)). The RF voltage had a frequency of 1.74 MHz and had an amplitude up to 3.6 kV peak‐to‐peak, coming from an LC tank that resonates at a frequency set by the capacitance of the QMF and an external, configurable inductance, resulting in a maximum resolvable mass of 250 Da for this configuration.

The sensitivity of the 3D‐printed quadrupoles was also characterized using the same experimental setup. The sensitivity, in mA/Torr, measures how much ion current the QMF transmits for a given background pressure and emission current at the ionizer while the quadrupole is tuned to unit mass resolution. In commercial quadrupole systems, the sensitivity is typically measured using nitrogen (28 Da) while the emission current is set to 2 mA. In this study, argon (40 Da) was used as the test compound, and a 0.5 mA emission current was used due to the limitations of the laboratory hardware. Assuming linearity between the emission current and transmitted current under 2 mA (this is in agreement with the electron‐impact ionization model^[^ ^22^ ^]^), the current transmitted by the QMF in the experiment is multiplied by four to estimate the quadrupole sensitivity.

**Figure 10:**
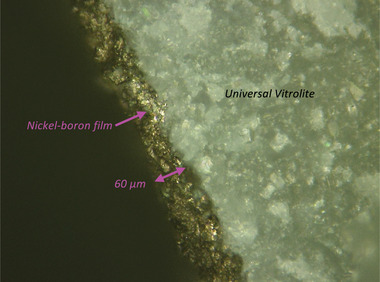
Optical micrograph of the edge of a 3D‐printed quadrupole rod coated by an electroless nickel‐boron film.

The thickness of the electroless nickel‐boron coating was measured using a laser scanning confocal microscope with nanometer resolution (Keyence VK‐X, Keyence, Itasca, IL, USA). The metal film thickness was found to be ≈60 ± 11 µm (**Figure** **10**), which is sufficient to contain the driving RF electromagnetic wave, as the skin depth of nickel at 1 MHz is ≈6 µm. Using the same instrument, the surface roughness (Ra) of the metal film was estimated at 6 µm, which is orders of magnitude larger than the roughness of commercial quadrupoles made of SS via precision machining and polishing (on the order of tens of millionths of an inch). The roughness in the metal film should create higher harmonics, as the shape of the rods is not exactly hyperbolic, which should degrade the device's resolution. However, devices with sharper features and electrode shapes farther from the ideal hyperbolic shape, e.g., quadrupoles with atomically sharp, square electrodes,^[^ ^23^ ^]^ are still able to show adequate performance, particularly when operated in the second stability region.

During electroless plating, the nickel–boron atoms required to metalize the 3D‐printed part are supplied by the flow of solution surrounding the surface to be coated in metal. To enhance the flow of species, one should both uniformly heat up (e.g., using a water bath) and stir (e.g., using a magnetic stirrer) the plating mixture; this way, the coating process is not supply‐limited and all surfaces should see the same deposition rate.^[^ ^24^ ^]^ In this regime, the thickness of the film deposited is time‐controlled and the deposition rate is linear if the bath is not depleted.^[^ ^25^ ^]^

Thermogravimetric analysis (TGA) of green, 3D‐printed vitrolite was conducted in a nitrogen atmosphere using a Discovery TGA (TA Instruments, New Castle, DE, USA) with a balance precision of 0.1 µg. For this analysis, the samples were heated between 35 and 800 °C at a rate of 20 °C min^−1^.

## 5 Results and Discussion

This section describes the characterization of the devices, which entailed three major thrusts: 1) exploration of their thermal performance, 2) exploration of the resolution and transmission of the QMFs, and 3) collection of mass spectrometry data.

### 5.1 Thermal Performance

**Figure 11:**
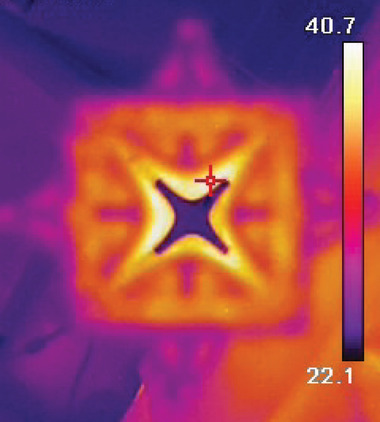
Infrared image of 3D‐printed QMF after running inside the vacuum chamber. The hotter regions of the devices correspond to the portions that are electrically conductive. In the picture, the emissivity was assumed to be equal to 0.9.

Despite their low electrical resistance and capacitance, the QMFs heat up during operation due to the lack of good heat transfer paths inside the vacuum chamber. To assess the thermal performance of the devices, a 3.6 kV peak‐to‐peak, 1.74 MHz resonant signal was applied to a quadrupole over an extended period of time (half an hour), during which no breakages or failures were observed. Right after venting the chamber, a process taking ≈5 min, the surface temperature of the quadrupole was recorded using a thermal camera (HIKMICRO B20, Hangzhou HIKMICRO Sensing Technology Co., Ltd, China), yielding a maximum temperature of 40.7 °C (**Figure** **11**). This temperature is representative of the temperature typically reached by the quadrupole after venting, following extended use, e.g., conducting mass spectrometry experiments. This temperature is a lower bound of the temperature attained by the QMF inside the vacuum chamber, as the part endures some convective cooling from the venting process. Nonetheless, the results show that the metallic surfaces of the QMF significantly heat up due to the RF wave; heat that is constrained to flow into the ceramic structure.

Figure 11 shows the temperature of the rods is not uniform; however, the temperature field has symmetry around an axis inclined −45° from the horizontal direction. Figure 11 is taken after venting the chamber; therefore, the nonuniformity in the temperature field might be due to the venting process. Nonetheless, the temperature nonuniformity could also be due to manufacturing issues in the dielectric structure or the metal coating.

**Figure 12:**
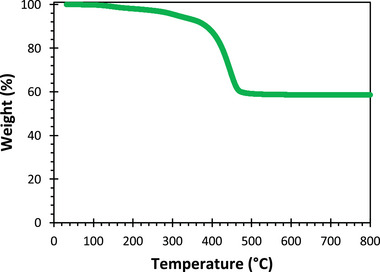
TGA data from green, 3D‐printed Universal Vitrolite in nitrogen using a ramp rate of 20 °C min^−1^.

The TGA data of 3D‐printed Universal Vitrolite show the material is remarkably stable across a wide range of temperatures (**Figure** **12**). At 150 °C, the material loses less than 1% of its mass—likely due to the release of moisture as the test is conducted in an inert atmosphere. It is not until ≈400 °C that the polymer sublimates out of the printed material, leaving the material composed exclusively of ceramic aggregates. The polymer sublimation is completed at ≈468 °C. After that, no other changes are recorded. Printable Vitrolite can be annealed at 900 °C with no issue; however, in a previous study, we found that 3D‐printed and annealed Vitrolite has three times the dimensional error of green counterparts.^[^ ^19^ ^]^

### 5.2 Mass Filtering Performance Optimization

**Figure 13:**
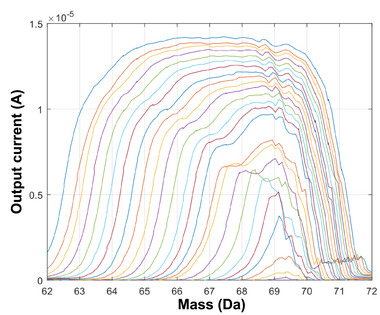
Spectral data from varying the resolving DC voltage while driving the QMF with RF.

**Figure 14:**
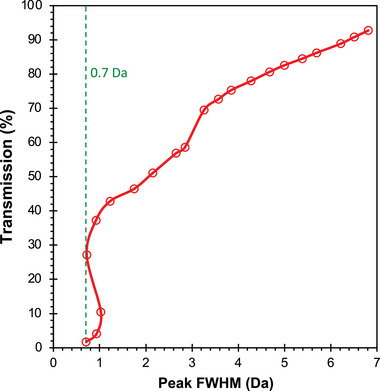
Transmission versus full width at half maximum (FWHM) of the data is shown in Figure 13.

The performance of a QMF at various mass‐to‐charge ratios can be benchmarked by the resolution, *R*, equal to

(4) $$R\;=\frac{M}{\Delta M}\;$$

where *M* is the peak mass and Δ*M* is the full width at half maximum (FWHM). Achieving optimal filtering performance in a QMF requires careful optimization of certain parameters, with a particular focus on the level of the resolving DC voltage applied to the power RF signals driving the quadrupole. The amount of resolving DC provided significantly impacts the performance of the quadrupole^[^ ^26^ ^]^: when insufficient resolving DC is applied, the transmission of ions through the quadrupole remains high, but the resulting resolution of the mass spectrum is compromised; conversely, excessive resolving DC leads to improved resolution but reduced ion transmission. To strike the right balance, the resolving DC levels of quadrupole filters are typically fine‐tuned to an intermediate value that aims to achieve the highest possible mass resolution while still maintaining sufficient ion transmission. By finding this optimal point, the quadrupole mass analyzer can effectively produce peaks with improved resolution and sensitivity, leading to more accurate and reliable mass spectra. To assess the performance of the additively manufactured quadrupoles, a device is first run using RF‐only voltage sweeps. This generates an RF‐only spectrum characterized by broad peaks, with their tails positioned near the actual mass‐to‐charge ratios of the test species’ characteristic mass peaks. After that, to refine and optimize the spectral resolution, the resolving DC voltage is gradually introduced to the driving voltage signal, effectively narrowing the peaks, and centering them more accurately around their true mass‐to‐charge ratios (**Figure** **13**). By compiling the results of a single mass peak obtained at various DC levels (in these experiments the 69 Da peak from FC‐43 was used), an approximate resolution‐transmission curve can be created. From this exercise, a minimum FWHM of 0.7 Da at 69 Da for 30% transmission is obtained (**Figure** **14**). The relative smoothness of these evolved peak shapes bodes well for accuracy of the quadrupolar fields in this unit, with a slight shoulder growing on the upper low mass side of the mass peak, with its relative peak position moving as mass resolution is increased, and a similar slight dimpling just to the right of the evolved mass peak, are indicative of octopolar field imperfections, attributed to symmetric errors in the overall quadrupolar field, as opposed to specific misalignment issues. These minor features in the peak shape do not dramatically affect the ability to identify and quantify mass peaks, and indeed are present in varying degrees in most commercial quadrupoles, especially if they are constructed from round rods. The absence of a satellite peak (pre‐cursor) just to the left of the mass peak indicates the absence of odd‐order (hexapolar and dodecapolar) field imperfections in the 3D‐printed quadrupoles.

### 5.3 Mass Spectrometry Data

**Figure 15:**
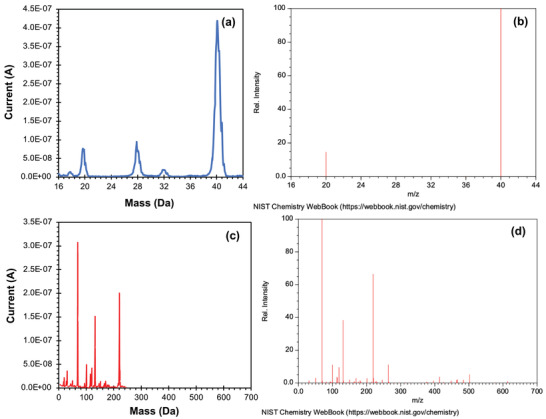
Mass spectra of argon a) using a 3D‐printed QMF and b) from NIST's database. Mass spectra of FC‐43 c) using a 3D‐printed QMF and d) from NIST's database.

**Figure 16:**
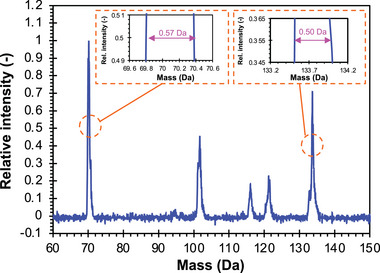
High‐resolution mass spectrum of FC‐43 from a 3D‐printed QMF.

Using an additively manufactured QMF calibrated for high transmission, mass scans were taken of argon and FC‐43, separately (**Figure** **15**). The data closely follow the expected peaks. For instance, the mass spectrum taken with argon shows clear peaks at 20 and 40 Da, representing Ar^+^ and Ar^2+^, respectively. Meanwhile, the data show traces of air inside the vacuum chamber, with peaks indicating the presence of nitrogen (14 and 28 Da), oxygen (16 and 32 Da), and water (17 and 18 Da). Furthermore, the FC‐43 shows the characteristic peaks of the compound in addition to smaller, trace peaks; there is a ≈1 Da shift between the measured mass peaks and the expected mass peaks. These results bode very well for the performance of the 3D‐printed quadrupoles. A quadrupole was also calibrated to maximize its mass resolution using FC‐43 (**Figure** **16**). The maximum resolution is equal to 121 for the 69 Da peak and 262 for the 131 Da peak. Therefore, our additively manufactured quadrupole can reach nominal unity mass resolution with these calibration settings. It is important to note that the mass range a QMF can reach is a product of the rod diameter, the capabilities of the driving electronics, and the maximum safe potential of the quadrupole in a vacuum (limited by arcing or material breakdown). Larger quadrupoles will have less mass range for a given RF driving voltage, and as such, our larger quadrupole, limited by the driving electronics utilized, had an experimental mass range of 250 Da. There is no indication, unlike other prior work, that material breakdown is a concern with our choice of materials; the limited mass range of this quadrupole is a direct function of its driving electronics.

**Table** **1** summarizes reported, miniaturized QMFs and compares them with this work and a commercial mass spectrometer with a QMF (Ardara Technologies, Ardara, PA, USA). The table includes studies that reported 3D‐printed, compact quadrupoles run as linear ion traps^[^ ^36^ ^]^ to illustrate the additively manufactured structures that have been proposed and studied. While there has been extensive work in recent years applying additive manufacturing techniques to quadrupole ion trap assemblies with respectable performance, quadrupole ion traps utilize different operational modes and cannot be used directly as mass filters; therefore, linear ion traps were not considered when comparing the performance attained in this work with the literature. The reported devices are made using silicon micromachining, which is expensive and time‐consuming, or via additive manufacturing. Also, most of the devices reported are not monolithic; to the best of our knowledge, the only other example of a compact, monolithic quadrupole is the work of Cheung et al.^[^ ^23^ ^]^

**Table 1:** Summary specifications and performance of reported, miniaturized QMFs. In the table *R* = resolution, i.e., *M*/Δ*M*, *AR* = aspect‐ratio, i.e., *L*/2*r_o,_* . Sensitivities are reported in mA/Torr from a 2 mA emission source. The commercial quadrupole reported is made by Ardara Technologies and run using their system. The QMFs were operated in the first stability region unless noted (SSR = second stability region).

Refrences	Manufacturing technology	Max. *R* (−)	*r*_o_ (µm)	*L* (mm)	*AR* (−)	*f* (MHz)	*R*/*AR* (−)	Sensitivity	Mass Range (Da)
[27]	Silicon micromachining, assembled rods	32 @ 40 Da	217	30	69.1	6	0.46	1.15 μA Torr^−1^	50
[28]	Silicon micromachining, assembled rods	36 @ 69 Da	690	90	65.2	1.44	0.55	8.01 nA Torr^−1^	650
		70 @ 28 Da	690	90	65.2	2	1.07		
		18 @ 69 Da	435	37	42.5	4	0.42		
[29]	Silicon micromachining, assembled rods	200 @ 219 Da	217	30	69.1	6	2.89	N/A	400
[23]	Silicon micromachining, monolithic rods	19 @ 69 Da	707	30	21.2	1.8	0.90	N/A	650
		40 @ 40 Da	707	30	21.2	4	1.89		50 @ SSR
		90 @ 28 Da	707	30	21.2	2	4.24		
[13]	3D‐printed, assembled rods	45 @ 18 Da	500	70	70.0	6	0.64	17.3 nA Torr^−1^	150
[14]	3D‐printed, assembled rods	11 @ 28 Da	870	70	40.2	3.5	0.27	N/A	55
[12]	3D‐printed, assembled rods	23 @137 Da	2500	40	8	0.985	2.85	N/A	Non‐scanning
^[^ ^30^ ^]^ Ion trap	3D‐printed, assembled rods	116 @ 175 Da 148 @ 349 Da	5000	25	2.5	0.73	175	N/A	350
[31]	3D‐printed, assembled rods	69.45 @ 4 Da	2000	50	12.5	3.686	5.56	66.7 nA Torr^−1^	10
^[^ ^32^ ^]^ Ion trap	3D‐printed, assembled rods	260 @ 398 Da	3000	40	6.66	1.185	39.04	N/A	1900
[33]	Metal rods, silicon assembly	140 @ 219 Da	325	30	46.2	6.5	3.03	N/A	1200
^[^ ^34^ ^]^ (eval. Of^[^ ^33^ ^]^)	Metal rods, silicon assembly	270 @ 242.47 Da	325	30	46.2	6.5	5.84	N/A	400
[35]	Silicon on glass rods	150 @ 219 Da	1000	45	22.5	N/A	6.67	N/A	800
This work	3D‐printed, monolithic rods	121 @ 69 Da 262 @ 131 Da	6000	125	10.4	1.74	11.04	0.13 mA Torr^−1^	250
Commercial quadrupole	Precision subtractive manufacturing, assembled rods	400 @ 69 Da	3000	200	16.7	1.7	24.00	0.6 mA Torr^−1^	10 000

The resolution of the 3D‐printed QMF reported in this study surpasses that of most of the other reported miniaturized devices and matches the work of Wright and colleagues.^[^ ^34^ ^]^ However, their data was obtained at 6.5 MHz, while our data was obtained at 1.74 MHz and *R*∝*f* ^2^.^[^ ^27^ ^]^ In principle, driving the 3D‐printed QMFs at higher frequency should increase the resolution; this would require increasing the RF and DC voltages so that the dimensionless parameters of the Mathieu equation would stay constant (Equations 2 and 3). However, driving the quadrupole at a higher frequency (hence higher voltages) will increase the heat losses. Although Vitrolite can be heated up to 900 °C, and no sublimation of polymers is perceived before ≈400 °C (Figure 12), this is a matter that needs to be further studied, as it might be possible that the difference in thermal expansion of the metal film and the ceramic structure could affect the operation of the devices. We can also see that the 3D‐printed QMFs reported in this study reach a high resolution despite their relatively low aspect ratio *AR*. Moreover, the dimensionless parameter *AR*/*R* groups the 3D‐printed QMFs reported in this work a lot closer to the commercial QMF. Nonetheless, our 3D‐printed QMFS are a lot cheaper (10 s of US dollars in materials) and faster (≈1 day) to make, showing a great potential for low‐cost, capable MS systems. There are several possibilities to increase the resolution of the 3D‐printed QMFs reported in this study. First, the RF frequency can be increased, e.g., operating the devices at ≈3.5 MHz would yield resolution on par with the commercial quadrupole @ 69 Da if the voltages were augmented accordingly. However, as explained before, operating the quadrupoles at larger frequencies and voltages will increase heating, which could affect the operation of the devices. Also, it should be possible to increase the performance of the 3D‐printed QMFs by making the devices longer, as *R*∝*L* ^2^.^[^ ^27^ ^]^ We demonstrated the longest QMFs that can be printed vertically in the Bison 1000; therefore, increasing the quadrupole length would require a different printer, a modification of the current printer, or a clever orientation of the object with respect to the platform. It might also be possible to make the separation between opposite rods smaller, resulting in a larger aspect ratio. However, this modification might adversely impact the resolution because the features of the QMF might not be defined as precise given the size of the voxels (57 µm× 57 µm× 100 µm). Furthermore, reducing the size of the aperture of the QMF should adversely impact its sensitivity.

For a partial pressure of 3 × 10^−5^ Torr Ar, a Faraday ion current of 9 nA was measured for 40 Da with a FWHM of 1 Da, with a 0.5 mA electron emission current. Normalizing to 2 mA electron emission current as commonly reported by commercial residual gas analyzer (RGA) manufacturers (e.g., Extorr Inc., New Kensington, PA, USA), the absolute sensitivity of the 3D‐printed quadrupoles is estimated at 0.13 mA Torr^−1^; this is only about a fourfold lower than typical commercial values (0.6 mA Torr^−1^)—it is encouraging that the sensitivity of the proof‐of‐concept, additively manufactured QMF is within an order of magnitude of optimized, commercial counterparts. Furthermore, the sensitivity of the 3D‐printed quadrupoles greatly surpasses the reported sensitivities of compact quadrupoles. This is not surprising, given that, if the emissive area of the ion source is larger than the acceptance of the quadrupole, the transmission of a quadrupole scales with rod diameter to the third or fourth power^[^ ^17^ ^]^ (the aperture of the 3D‐printed quadrupoles is comparable to that of a commercial quadrupole). Miniaturizing a quadrupole mass filter reduces its sensitivity, due to both the reduction of the inlet aperture and the manufacturing precision.^[^ ^17^ ^]^ Therefore, there is a trade‐off between miniaturization and the usefulness of the quadrupole. This study aimed at implementing the most compact quadrupoles that would still be useful for mass spectrometry.

The mass range demonstrated with the 3D‐printed quadrupoles (250 Da) is smaller than that reported in many studies,^[^ ^23^, ^28^, ^29^, ^30^, ^32^, ^33^, ^34^, ^35^ ^]^; furthermore, commercial mass spectrometers typically deliver 1000s Da of mass range (Table 1,^[^ ^37^ ^]^). However, the mass range of the 3D‐printed QMFs is adequate for RGA applications, which typically cover between 100 and 300 Da. The mass range of a quadrupole is directly related to the diameter of its rods (for a given frequency, a larger rod diameter requires a larger voltage to have stable ion trajectories within the quadrupole), the capabilities of the driving electronics, and the maximum safe potential of the quadrupole in vacuum (limited by arcing or material breakdown). The reported mass range of the 3D‐printed QMFs is limited by the maximum RF voltage available to drive the quadrupoles (3.6 kV), while in many cases in the literature, the maximum voltage used reflects the electrical breakdown voltage of the quadrupoles (this is typical in devices electrically insulated with thin‐film dielectrics, e.g.,^[^ ^23^, ^27^, ^28^ ^]^). Nonetheless, using larger bias voltages to drive the QMF should result in larger heat losses and higher temperatures of operation, which could adversely influence the performance of the quadrupoles. Reducing the inscribed radius of the quadrupole could readily increase mass range, at the expense of decreasing mass resolution (due to poorer relative mechanical precision) and a dramatic reduction in absolute sensitivity.

### 5.4 Future Work

This study reports a proof‐of‐concept demonstration of monolithic, 3D‐printed quadrupole mass filters that are both less expensive and more compact than commercial counterparts, with adequate resolution to conduct mass spectrometry. We identify several directions for extending the work conducted in this study:

- It might be possible to implement 3D‐printed QMFs that transfer better heat if they are made of a more suitable ceramic, e.g., boron nitride. This requires further research and development of printable materials and printing technology, developing additive manufacturing processes for novel materials without sacrificing already satisfactory aspects of the manufacturing such as printing resolution and outgassing.
- It would be interesting to demonstrate an integrated instrument. To develop such an instrument, the time and temperature stability of the 3D‐printed quadrupoles should be characterized and controlled. As a matter of fact, there are vendors (e.g., Agilent, Bruker) that implement temperature control in their quadrupoles to ensure the stability of the instrument. This would be facilitated by implementing mass filters with better heat transfer. The work would include characterizing the thermal properties of the printed materials such as thermal expansion, heat capacity, and thermal conductivity, to be able to conduct simulations to guide the device design. A possibility for reducing the thermal losses is to coat the quadrupole electrodes with metal films that have significantly smaller electrical resistivity, e.g., gold. As a matter of fact, there are commercial quadrupoles with gold‐plated silica rods (e.g., Agilent GCMS).
- It would be interesting to demonstrate an integrated instrument made as much as possible via additive manufacturing. The cost of a mass spectrometer is not dominated by the cost of the mass filter; nonetheless, the cost of the mass filter is significant (it is ≈10–15% of the overall cost of manufacturing of a quadrupole mass filter without including the pumps). As a comparison, the manufacturing of the 3D‐printed quadrupoles takes about a day (including metallization) and costs tens of dollars; this is in strike difference with the cost of a commercial quadrupole (thousands of dollars) that typically requires integration of components from multiple vendors and whose individual component manufacturing processes can take weeks to complete. Other key components of the mass spectrometer, e.g., ionizer ^[^ ^38^, ^39^ ^]^, and vacuum chamber^[^ ^40^ ^]^ have been demonstrated to properly work when made via 3D printing; manufacturing them also incurs a small fraction of the cost of commercial counterparts. Therefore, implementing a mass spectrometer made as much as possible via 3D printing should yield a significantly cheaper instrument. This is not surprising, as 3D printing has been shown to manufacture faster and cheaper small‐batch to mid‐batch complex devices.^[^ ^41^ ^]^

## 6 Conclusion

This study reported the proof‐of‐concept demonstration of compact, additively manufactured QMFs with competitive filtering performance for mass spectrometry applications. The QMFs are monolithically fabricated via vat photopolymerization of the glass–ceramic Universal Vitrolite and selective electroless nickel‐boron plating. Experimental characterization of QMF prototypes at 1.74 MHz using FC‐43 yields 131 Da peaks with 0.50 Da full width at half maximum (262 resolution), surpassing the resolution of reported miniaturized counterparts and approaching the performance standards of commercial, non‐miniaturized devices. The 3D‐printed QMFs have a sensitivity of 0.13 mA Torr^−1^, which is comparable to that of commercial counterparts and larger than the sensitivity of reported miniaturized quadrupoles. The devices attained a mass range of 250 Da, limited by the electronics, and smaller than many of the reported miniaturized quadrupoles. Proposed future work includes developing a 3D printing process for ceramics with higher thermal conductivity (e.g., boron nitride), characterizing and controlling the time and temperature stability of the quadrupoles (of great importance in an integrated instrument), and demonstrating an integrated mass spectrometer with more 3D‐printed components (e.g., ionizer, vacuum chamber).

The use of additive manufacturing in creating a monolithic QMF marks a significant advancement in the field. This innovative approach has yielded a quadrupole mass analyzer with competitive filtering performance while being efficient in cost and size. Despite potential material improvements, our design methodologies and the validation of the achieved results should serve as a source of motivation for future endeavors in leveraging additive manufacturing techniques for mass spectrometry and other scientific instruments. The successful fabrication and performance validation of our additively manufactured quadrupole demonstrates the feasibility and potential of this manufacturing approach in advancing the capabilities of mass spectrometry instruments. The combination of innovative design strategies, new materials, and empirical evidence substantiating the efficacy of additively manufactured components in fulfilling demanding requirements paves the way for exciting possibilities and advancements in this interdisciplinary field.

## Conflict of Interest

The authors declare no conflict of interest.

## Author Contributions

C.C.E. performed data curation, formal analysis, investigation, methodology, software, validation, and visualization, and wrote, reviewed, and edited the original draft. N.K.L. performed Investigation, methodology, wrote, reviewed, and edited. L.J.M. Methodology, wrote, reviewed, and edited. R.E.P. Formal analysis, Methodology, wrote, reviewed, and edited. L.F.V.‐G. Conceptualization, Funding Acquisition, Methodology, Project Administration, Resources, Supervision, wrote, reviewed, and edited the original draft.

## Data Availability

The data that support the findings of this study are available from the corresponding author upon reasonable request.
